# Analysis of Volatile Organic Compounds in Wines from *Vitis amurensis* Varieties in Xinjiang, China

**DOI:** 10.3390/foods14203521

**Published:** 2025-10-16

**Authors:** Yining Sun, Mengqi Wang, Weiyu Cao, Mingjie Ma, Peilei Xu, Changyu Li, Yue Pan, Wenpeng Lu

**Affiliations:** 1 Institute of Special Animal and Plant Sciences, Chinese Academy of Agricultural Sciences, Changchun 130112, China; 82101225210@caas.cn (Y.S.); 82101232246@caas.cn (M.W.); 82101231147@caas.cn (W.C.); xupeilei@caas.cn (P.X.); lichangyu@caas.cn (C.L.); 2 College of Agriculture, Yanbian University, Yanji 133002, China; 2023050869@ybu.edu.cn; 3 Xinjiang Uygur Autonomous Region Academy of Forestry Sciences, Urumqi 830092, China

**Keywords:** wine, HS-GC-IMS, OAV, E-nose, multivariate statistical analysis

## Abstract

As a wine-producing region in China, Xinjiang’s ecological conditions endow grapes with distinctive flavor potential. However, systematic research on volatile compounds in wines from *Vitis amurensis* Rupr. varieties in this region remains limited. Therefore, wines from four Xinjiang *Vitis amurensis* varieties (‘Shuanghong’, ‘Zuoyouhong’, ‘Xuelanhong’, and ‘Beibinghong’) were analyzed using high-performance liquid chromatography (HPLC), headspace gas chromatography–ion mobility spectrometry (HS-GC-IMS), electronic nose (E-nose), odor activity value (OAV) calculation, and multivariate analysis. Physicochemical parameters, organic acids, volatile organic compounds (VOCs), and OAVs were determined. Results showed significant differences in physicochemical properties among the varieties, potentially correlating with wine mouthfeel. Beibinghong wine contained the highest total VOC concentration. Among 64 identified VOCs, 37 had OAVs ≥ 1. Multivariate analysis identified 14 key differential volatile compounds (VIP ≥ 1, *p* < 0.05) responsible for flavor differences between varieties, with each variety exhibiting distinct key compounds. E-nose analysis effectively distinguished the aroma profiles of the four wines. This study elucidates the chemical and volatile compound characteristics of wines from Xinjiang *Vitis amurensis* varieties, providing a theoretical foundation for research on their flavor profiles. It also aids in selecting *Vitis amurensis* varieties for cultivation and supports the development of distinctive regional wines in Xinjiang.

## 1. Introduction

Wine is a beverage widely favored by consumers [1], and aroma serves as a core factor determining its quality and consumer preference. Varietal characteristics, winemaking techniques, terroir, and aging processes all influence a wine’s aromatic profile [2,3]. For instance, studies from Australia, Spain, Turkey, and the Bordeaux and Provence regions of France demonstrate that distinct regions yield unique primary aroma compound profiles in rosé wines [4]. Comparative studies of the Amyndeon and Naoussa regions in Greece further demonstrate that terroir differences directly determine aroma typicity by regulating grape constituents (nitrogen, lipids) and metabolic pathways [5].

To better distinguish the aromatic characteristics of wine, modern instrumental analysis techniques play a crucial role. Headspace Gas Chromatography–Ion Mobility Spectrometry (HS-GC-IMS) achieves highly sensitive detection of trace volatile organic compounds (VOCs) through a dual separation mechanism (column separation and ion mobility time separation). This technique excels in resolving aroma-active compounds within isomers and complex matrices, enabling the construction of VOC fingerprint profiles [6]. The Electronic Nose (E-nose) employs pattern recognition technology based on holistic sensor array responses to aromas. Through algorithms such as Principal Component Analysis (PCA) and Linear Discriminant Analysis (LDA), it establishes discriminant models based on E-nose data to rapidly distinguish wine aroma profiles [7]. These techniques are now widely applied in wine aroma identification [8].

China’s vast territory and diverse terroirs have given rise to typical wine-producing regions such as Shandong, Ningxia, and Xinjiang. Among these, Xinjiang—as China’s earliest grape-growing region and the birthplace of wine—possesses advantageous natural conditions including soil, diurnal temperature variation, and sunlight exposure. It holds significant potential for producing high-quality wines with distinct regional characteristics [9]. In recent years, Xinjiang’s wine industry has experienced rapid development. Four major wine regions—the Northern Foothills of the Tianshan Mountains, Ili, Yanqi, and Hami—have been developed and certified [10].

Research on wine regions focuses on two aspects: first, the variation in flavor characteristics caused by terroir differences across regions for the same grape variety—for example, Cabernet Sauvignon wines exhibit distinct volatile compounds and aromatic profiles among seven sub-regions of Shangri-La [11]; while the Hongsibao sub-region in Ningxia’s Helan Mountain East Foothills shows pronounced floral notes and the Qingtongxia sub-region displays green spice tones [12]. Second, the analysis of flavor characteristics of different grape varieties within the same region. The Weibei Plateau region compared six varieties (Granoir, Dunkelfelder, Meili, Moldova, Gongniang, Beibinghong) and their wines regarding physicochemical properties, phenolic compounds, organic acids, and aroma components [13]. Xinjiang explored the flavor profiles of Cabernet Sauvignon, Cabernet Franc, Marselan, Merlot, Malbec, Syrah, Petit Verdot, Dornfelder, and Pinot Noir [14]. However, most studies focus on widely cultivated varieties, with limited research on other resource varieties. For instance, the wild grape species *Vitis amurensis* Rupr. in Xinjiang possesses cold tolerance and unique flavor compounds, attracting winemaking interest for its potential to impart distinctive characteristics to wines [15]. Varieties such as ‘Shuanghong’, ‘Zuoyouhong’, ‘Xuelanhong’, and ‘Beibinghong’ have been developed and cultivated in Xinjiang [16,17]. Systematic research on the aromatic characteristics of wines from these newly introduced *Vitis amurensis* varieties in Xinjiang remains scarce. It is particularly unclear whether different varieties can exhibit unique, distinguishable aromatic profiles under identical terroir conditions.

We hypothesized that under identical terroir and winemaking conditions in Xin-jiang, different wild grape varieties would develop distinct overall aroma pro-files and VOC fingerprint patterns due to genetic differences. To validate this hypothesis, wines from four *Vitis amurensis* Rupr. varieties—‘Shuanghong’, ‘Zuoyouhong’, ‘Xuelanhong’, and ‘Beibinghong’—were studied. A combined approach of E-nose and HS-GC-IMS technologies, integrated with conventional physicochemical analysis, was used to systematically characterize the volatile compound fingerprints of these four Xinjiang *Vitis amurensis* wines for the first time, identifying key differentiating compounds between varieties. This research fills a gap in flavor studies of Xinjiang wild grape varieties and provides scientific support for optimizing wine quality and developing distinctive products.

## 2. Materials and Methods

### 2.1. Grape Sampling and Winemaking

The experimental site was located at the Jiamu Long-term Scientific Research Base for Pomology in Wensu County, Aksu Region, Xinjiang (latitude 41°15′ N, longitude 80°32′ E). The region experiences a temperate arid climate, with an average annual precipitation of less than 100 mm; the mean annual temperature is 10.1 °C, the average annual precipitation is 65.4 mm, and the average annual frost-free period is 185 days. The tested grape varieties were ‘Beibinghong’, ‘Shuanghong’, ‘Zuoyouhong’, and ‘Xuelanhong’. The vines were planted at a spacing of 1 m × 3.5 m along north–south rows, trained in a T-shaped system with a vertical canopy. Field management, including irrigation and fertilization, followed standard practices, and all sampled vines exhibited consistent growth vigor.

Grapes from four varieties (‘Beibinghong’, ‘Shuanghong’, ‘Zuoyouhong’, and ‘Xuelanhong’) were harvested in September 2023. The experiment was set up as a com-pletely randomized design (CRD) with three biological replicates per variety. After harvest, grapes were sorted to remove damaged fruit, destemmed, crushed, and transferred to fermentation tanks. Room temperature was maintained at 25–28 °C. Food-grade potassium metabisulfite was added at 105 mg/L, followed by inoculation with *Saccharomyces cerevisiae* strain CEC01 (Angel Yeast Co., Ltd., Yichang, China) at 250 mg/L. During primary fermentation, tanks were sealed with air release valves to allow gas venting. Fermentation completion was determined by monitoring total sugar content; primary fermentation concluded when sugar levels stabilized. Subsequently, lees were separated, and the supernatant underwent secondary fermentation at 18–20 °C for one month. This phase assessed physicochemical indicator variations; since indicators remained stable, wines proceeded to analysis [18]. Sample numbers are listed in Table 1.

### 2.2. Chemical Reagents

Sodium hydroxide, anhydrous sodium carbonate, anhydrous sodium acetate, ferrous sulfate, sulfuric acid, hydrochloric acid (Beijing Chemical Plant, Beijing, China); Anthrone, potassium hydrogen phthalate, aluminum nitrate nonahydrate, potassium chloride, potassium sodium tartrate, resorcinol, methanol, anhydrous ethanol, phenolphthalein (Sinopharm Chemical Reagent Co., Ltd., Shanghai, China); Sodium acetate, anhydrous glucose (Xilong Chemical Co., Ltd., Shantou, China); Tannic acid, gallic acid standard (Tianjin Guangfu Fine Chemical Research Institute, Tianjin, China); Folin-Denis reagent (Feijing Biotechnology Co., Ltd., Fuzhou, China); Methanol (chromatographic grade, Tianjin Concord Technology Co., Ltd., Tianjin, China); Folin-Denis reagent, tartaric acid, malic acid, lactic acid, acetic acid, anhydrous citric acid, succinic acid (Shanghai Yuanye Biotechnology Co., Ltd., Shanghai, China); 4-Methyl-2-pentanol (Shanghai Lianshuo Biotechnology Co., Ltd., Shanghai, China).

### 2.3. Determination of the Chemical Indicators Parameters of Wines

All routine physicochemical parameters of wine were measured in triplicate. Specific methods are as follows. Total acidity: Determined by acid-base titration according to GB/T 15038-2006 [19], expressed as tartaric acid equivalents.

Total sugar: Quantified by anthrone–sulfuric acid colorimetry. This is the standard curve for glucose:

(1) y = 3.7136x + 0.1229, R^2^ = 0.9947

y represents measured absorbance and x represents total sugar content of the sample (mg/L). Samples reacted with anthrone–sulfuric acid reagent in a boiling water bath for 10 min, cooled, and absorbance measured at 625 nm.

Tannin content: Determined by Folin-Denis method. This is the standard curve for tannic acid:

(2) y = 0.3953x + 0.0531, R^2^ = 0.9908

y represents the measured absorbance and x represents the tannin content of the sample (g/L). Samples mixed with Folin-Denis reagent and saturated sodium carbonate solution, incubated at 85 °C for 30 min, cooled, and absorbance measured at 740 nm.

Total phenolics: Quantified by Folin–Ciocalteu method. This is the standard curve for gallic acid:

(3) y = 0.0069x + 0.0663, R^2^ = 0.9923

y represents the measured absorbance and x represents the total phenolic content of the sample (mg/L). Folin–Ciocalteu reagent added to samples, reacted for 5 min, followed by 20% sodium carbonate solution, incubated in dark at room temperature for 60 min, and absorbance measured at 765 nm. Results expressed as gallic acid equivalents.

Total anthocyanins: Determined by pH differential method. Samples diluted with pH 1.0 KCl buffer (0.025 M) and pH 4.5 NaOAc buffer (0.4 M), equilibrated at room temperature for 30 min, absorbance measured at 520 nm and 700 nm. Results expressed as cyanidin-3-glucoside equivalents [20].

### 2.4. Determination of Organic Acid Compounds

Organic acids in wine were identified and quantified by high-performance liquid chromatography (1200 Infinity II, Agilent Technologies, Waldbronn, Germany) according to Method [21]. Analysis used an Agilent C18-XT column (250 mm × 4.6 mm i.d., 5 μm particle size). The mobile phase consisted of Solvent A (0.1% phosphoric acid aqueous solution, pH 2.3) and Solvent B (methanol) in a 97:3 ratio, with a flow rate of 0.4 mL/min. Column temperature was maintained at 25 °C, and injection volume was 10 μL. Detection occurred at 210 nm, with each sample tested in triplicate. Organic acids were identified by retention time comparison with standards. Quantification used a standard curve correlating concentrations with peak areas. The standard curve, R^2^, and linear range are provided in Supplementary Materials Table S1.

### 2.5. Determination of Aroma Compounds

#### 2.5.1. HS-GC-IMS Analysis

Volatile compounds in wine were analyzed using a FlavourSpec^®^ flavor analyzer (G.A.S. Gesellschaft für analytische Sensorsysteme mbH, Dortmund, Germany) according to Method [22]. A 1 mL wine sample and 20 μL internal standard (4-methyl-2-pentanol, 10 mg/L) were placed in a 20 mL headspace vial. The mixture was incubated at 60 °C and 500 rpm for 10 min, with three sample replicates. A 100 μL injection was performed at 85 °C using an MXT-WAX IMS column (30 m × 0.53 mm, 1 μm film thickness) with a column temperature of 60 °C. Separation occurred at an IMS detector temperature of 45 °C. Nitrogen (99.999%) served as both carrier and drift gas. The carrier gas program was: 2 mL/min for 2 min, increased to 100 mL/min over 8 min, then maintained at 100 mL/min for 10 min. Drift gas flow was 150 mL/min.

VOCs were identified in VOCal software (version 0.4.03) using database, NIST, and IMS libraries. Relative quantification compared peak areas with the 4-methyl-2-pentanol internal standard. Mass concentration was calculated as:

(4) $$Ci=\frac{Cis\times Ai}{Ais}$$

where *Ci* denotes the calculated mass concentration of the volatile organic compound (μg/L), Cis denotes the mass concentration of the internal standard (4-methyl-2-pentanol, μg/L), Ai corresponds to the peak volume of the compound, and Ais denotes the peak volume of the internal standard. The internal standard (4-methyl-2-pentanol) was formulated at a concentration of 198 μg/L with a peak volume of 259.64, and the intensity of each peak was approximately equal to 0.763 μg/L.

Based on volatile compound quantification, odor activity values (OAV) were calculated as:

(5) $$OAV=\frac{Ci}{OTi}$$

where *Ci* is the compound concentration (μg/L) and *OTi* is the odor threshold (μg/L). Odor thresholds were sourced from *Odour Thresholds*.

#### 2.5.2. E-Nose Analysis

Following the described method, analysis was performed using an electronic nose (PEN3, Airsense Analytics GmbH, Schwerin, Germany) [23]. For each sample, 1.0 mL of wine made from *Vitis amurensis* Rupr. grapes was transferred to a headspace vial and equilibrated for 30 min at 50 °C. Analyses were performed in triplicate. Operational parameters are listed in Table 2, and the sensor array composition is detailed in Supplementary Materials Table S2.

### 2.6. Statistical Analysis and Data Visualization

Data processing used Microsoft Excel 2016 for mean ± standard deviation calculations. Data were analyzed using one-way analysis of variance (ANOVA) appropriate for the completely randomized design, followed by Duncan’s multiple range test (*p* < 0.05) for mean separation. HS-GC-IMS data were processed with VOCal (version 0.4.03). SIMCA 14.1 conducted orthogonal partial least squares discriminant analysis (OPLS-DA) and VIP analyses; GraphPad Prism 10 generated bar charts; cluster heatmaps, radar charts, and PCA plots was performed using the Metware Cloud, a free online platform for data analysis (https://cloud.metware.cn, accessed on 16 March 2025).

## 3. Results and Discussion

### 3.1. Chemical Indicators of Wines

**Figure 1:**
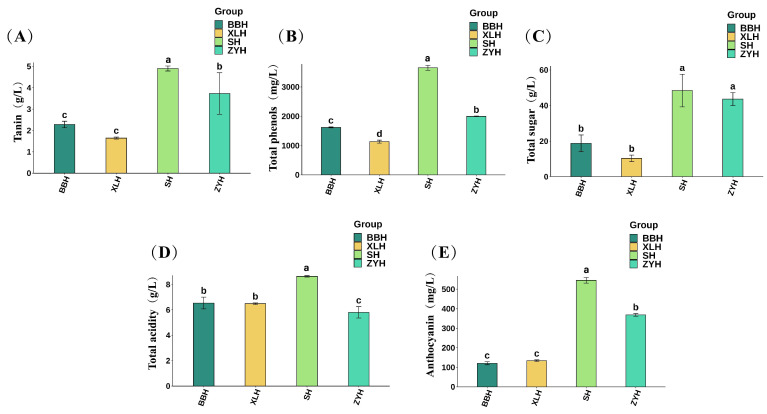
Physicochemical parameters of four wine varieties. (**A**) Tannin; (**B**) Total phenols; (**C**) Total sugars; (**D**) Total acidity; (**E**) Anthocyanin content. Different letters indicate significant differences (*p* < 0.05, Duncan’s test).

Chemical indicators are key determinants of wine quality [24]. Significant differences in chemical indicators occurred among the four grape varieties (Figure 1). Tannin content imparts astringency and body to wine [25]. Values ranged from 1.64 to 4.90 g/L, with SH exhibiting the highest tannin content and XLH the lowest. Total phenolic content enhances wine’s antioxidant capacity and enriches its flavor profile [26]. SH displayed the highest total phenolic content at 3654.27 mg/L. Compared to previous studies [18], the Xinjiang varieties exhibited higher total phenolic and tannin contents, potentially due to water stress from the hot, arid climate of Aksu, Xinjiang promoting greater accumulation of polyphenols and tannins [10].

Wines are classified as dry, semi-dry, sweet, or semi-sweet based on total sugar content [27]. BBH and XLH had total sugar content ≤4.0 g/L, classifying them as dry wines. SH and ZYH had total sugar contents of 4.85 g/L and 4.37 g/L, respectively, making them semi-dry wines. Total acidity ranged from 5.80 to 8.64 g/L, with SH exhibiting the highest and ZYH the lowest total acidity. Anthocyanins are the primary pigments responsible for wine color and influence sensory attributes [28]. Anthocyanin content decreased in the order: SH (545.40 mg/L) > ZYH (368.66 mg/L) > XLH (134.48 mg/L) > BBH (120.88 mg/L). Given consistent terroir, ripening periods, and winemaking processes, these differences are attributed to varietal characteristics.

### 3.2. Analysis of Organic Acid Content in Four Wine Varieties

**Figure 2:**
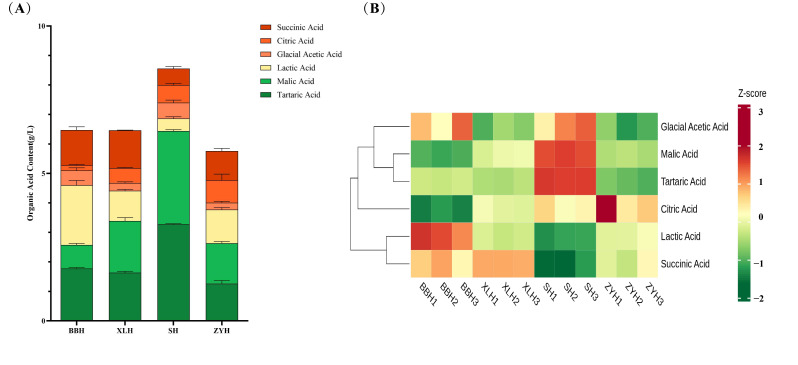
Overview of Organic Acids in Four Different Wine Varieties. (**A**) shows a bar chart of the six organic acids (tartaric acid, malic acid, lactic acid, glacial acetic acid, citric acid, succinic acid) in wine. (**B**) presents a cluster heatmap of the six organic acids’ concentrations in wine.

Organic acids are essential components of wine, influencing its overall palate harmony [9]. Six organic acids were identified in the wines of the four varieties: tartaric acid, malic acid, lactic acid, glacial acetic acid, citric acid, and succinic acid (Figure 2A). The composition and content of organic acids are largely determined by the grape variety’s genetic background [29], explaining the significant differences observed in these acids across the four wines. Tartaric, malic, and lactic acids were the predominant organic acids. Tartaric acid concentrations ranged from 1.26 to 3.28 g/L. Malic acid ranged from 0.80 to 3.16 g/L, with SH exhibiting the highest level. Lactic acid concentrations varied between 0.43 and 2.02 g/L; BBH, XLH, and ZYH showed significantly higher levels than SH. Lactic acid is primarily produced through malolactic fermentation (MLF). SH had the highest malic acid but the lowest lactic acid content, suggesting only partial MLF occurred [30]. SH also exhibited the highest cumulative organic acid concentration (8.56 g/L), followed by BBH, XLH, and ZYH (Figure 2A). Glacial acetic acid content was highest in SH and lowest in ZYH, while citric acid content was highest in ZYH (Figure 2B). Succinic acid content was higher in BBH and XLH than in SH and ZYH. The variation in organic acid types and concentrations influenced wine palate [31], further demonstrating their importance as indicators of wine quality.

### 3.3. Analysis of Volatile Compounds in Four Wine Varieties

#### 3.3.1. Volatile Compound Content Analysis

**Figure 3:**
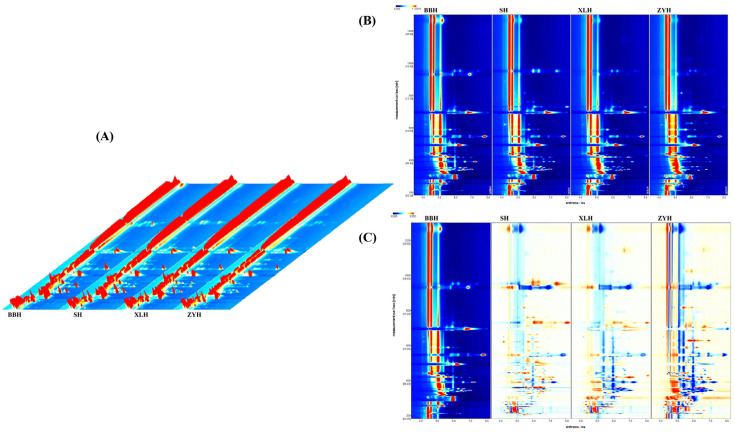

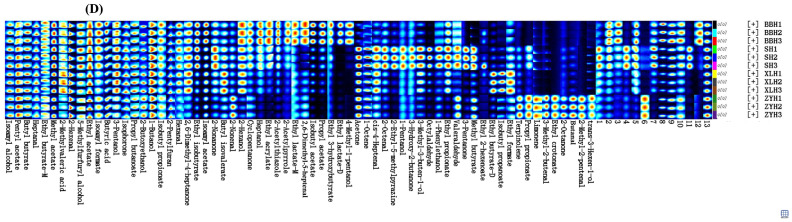
HS-GC-IMS volatile compound profile analysis of four different wine varieties. (**A**) Three-dimensional profile of volatile compounds from the four wine samples. (**B**) Two-dimensional profile of volatile compounds from the four wine samples. (**C**) Differential profile generated using BBH as the reference. (**D**) Fingerprint of volatile compounds from the four wine samples.

Using HS-GC-IMS technology, volatile compounds in four different wine samples were measured, resulting in Figure 3. Figure 3A. shows a three-dimensional spectrum generated by the reporting plugin in the analysis software. Red peaks represent volatile compounds, with peak height indicating peak volume and higher volatile compound concentrations. Figure 3B shows a two-dimensional map where each point represents a VOC. The distinct peak shapes among samples in Figure 3A and the varying colors of sample points in Figure 3B indicate differing volatile compound contents across the wine varieties. Using the BBH spectrum as a reference, the difference map for each sample is shown in Figure 3C. Colors indicate VOC concentrations: red denotes relatively high concentrations, white indicates low concentrations, and blue signifies concentrations below the detection limit. ZYH shows a significant increase in concentration compared to BBH, while SH and XLH exhibit only minor increases. The difference map further confirms variations among the four distinct wine varieties. Figure 3D presents the fingerprint spectrum, formed after qualitative analysis of volatile compounds in the samples. The spectrum identifies 77 signals: 64 known compounds and 13 unknown compounds. Among the known compounds, Ethyl butyrate and Ethyl lactate exist as dimers. Brighter spots in the fingerprint spectrum indicate higher signal intensities. Three parallel analyses of the four wine samples were performed, and distinct differences in volatile content were observed. BBH samples exhibited higher concentrations of volatiles such as 4-Methyl-1-pentanol, Ethyl lactate, Ethyl 3-hydroxybutyrate, Propyl acetate, and Isobutyl acetate compared to the other three samples. The SH sample exhibited higher concentrations of volatile compounds such as Isobutyl acetate, 3-Pentanone, Valeraldehyde, and 1-Phenylethanol compared to the other three samples. The XLH sample showed higher concentrations of volatile compounds including Ethyl formate, Isobutyl propanoate, and Ethyl butyrate-D compared to the other three samples. The ZYH sample exhibited higher concentrations of volatile compounds such as trans-3-Hexenol, 2-Methyl-2-pentenal, Butanal, and 2-Octanone compared to the other three samples, though precise quantitative analysis is required for specific content variations. The fingerprint spectra clearly demonstrate the distinctions in volatile compounds among different wine varieties; this phenomenon demonstrates that HS-GC-IMS can effectively separate and visualize these compounds in the four wine samples, validating its practicality in winemaking research.

**Figure 4:**
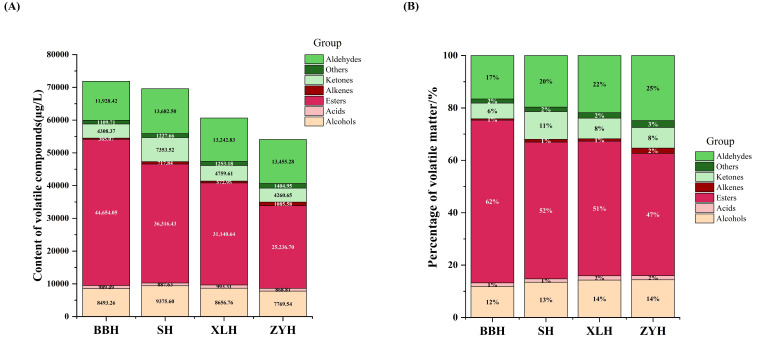
Volatile compound composition of four wine varieties. (**A**) Stacked bar chart of volatile compound concentrations. (**B**) Stacked bar chart of relative proportions (%) of volatile compound classes.

Through qualitative analysis, 64 volatile compounds were identified, comprising 23 esters, 12 alcohols, 12 aldehydes, 9 ketones, 3 alkenes, 2 acids, 1 pyrazine, and 2 other compounds. Quantitative analysis was performed based on qualitative results, with detailed data in Supplementary Material Table S3. As shown in Figure 4, total volatile compound concentrations in the four wines ranged from 54,187.37 to 72,111.42 µg/L, ranked as BBH > SH > XLH > ZYH. This aligns with previous findings that genetic backgrounds of grape varieties determine metabolic pathways, influencing aroma precursor types/levels and consequently volatile compound variations [13].

Esters constituted the highest proportion (46.57–61.92%), with BBH having the highest and ZYH the lowest content. Esters primarily form during fermentation; differences in grape juice composition among varieties may alter yeast metabolism, affecting ester synthesis [32]. Aldehydes ranked second (16.54–24.83%), highest in ZYH and lowest in BBH. As oxidation products of unsaturated fatty acids, some aldehydes react with polyphenols. Varietal differences in aldehyde content may arise from varying accumulation capacities of reactive polyphenols in wines [33]. Alcohols accounted for 7.085–8.76%, ketones for 5.89–6.96%, while alkenes, pyrazines, acids, and other compounds showed the lowest proportions.

#### 3.3.2. Multivariate Statistical Analysis

**Figure 5:**
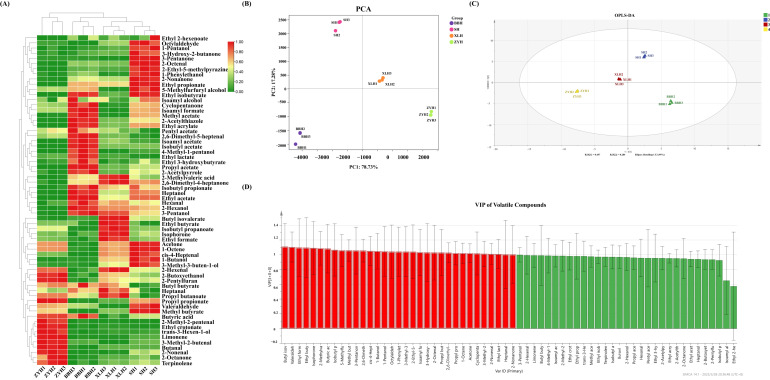
Multivariate statistical analysis of volatile compounds in four wine varieties. (**A**) Hierarchical clustering heatmap of volatile compound content (red: high; green: low; yellow: moderate). (**B**) PCA score plot. (**C**) OPLS-DA score plot. (**D**) VIP scores of volatile compounds.

Hierarchical cluster analysis (HCA) was performed on four wine varieties, with the resulting heatmap shown in Figure 5A. Volatile compound content is visualized by color: red indicates high content, green low content, and yellow moderate content. Samples clustered by variety, with ZYH forming a distinct group while BBH, SH, and XLH grouped together. PCA of the wines (Figure 5B) revealed clear varietal separation. The first two principal components (PC1 and PC2) explained 78.73% and 17.28% of variance, respectively, cumulatively accounting for 96.01% of total variance.

OPLS-DA was further applied using the 64 shared volatile compounds as dependent variables and the four varieties as independent variables. All samples fell within confidence intervals in Figure 5C, with triplicates per variety clustering tightly, confirming sample reliability. A permutation test (n = 200) validated the model, as the Q^2^ regression line intercepted the vertical axis below zero.

Thirty-three key differential volatile compounds were identified from the 64 shared compounds using VIP > 1 and *p* < 0.05 criteria (Figure 5D). These compounds form the material basis for inter-varietal aroma differences, providing a theoretical foundation for wine variety authentication and origin traceability through fingerprinting, indicating industrial application potential.

### 3.4. Analysis of Aroma Volatile Compounds in Four Wine Varieties

Wine aroma is primarily determined by volatile compounds, and differences among varieties are the main cause of diverse wine aromas. However, aroma perception is complex, depending not only on compound concentrations but also on their odor thresholds [34]. OAV is a key metric for evaluating individual compound contributions to overall aroma: OAV ≥ 1 indicates a key aroma contributor, while OAV < 1 suggests negligible contribution [35]. This study calculated OAVs to assess compound contributions. In the four Xinjiang wine varieties (BBH, SH, XLH, ZYH), 37 key volatile compounds with OAV ≥ 1 were identified, including esters (12), aldehydes (10), ketones (6), alcohols (4), alkenes (3), and others (1) (Table 3). These compounds form the typical aroma foundation of these varieties.

Esters are core components contributing to fruity and sweet aromas [36]. Ethyl isobutyrate, ethyl acetate, and ethyl butyrate exhibited exceptionally high OAVs (>1000). Among non-esters, cis-4-heptenal (aldehyde) also exceeded OAV 1000, primarily contributing dairy-like notes. These high OAVs indicate their significant roles in the aroma profile.

**Figure 6:**
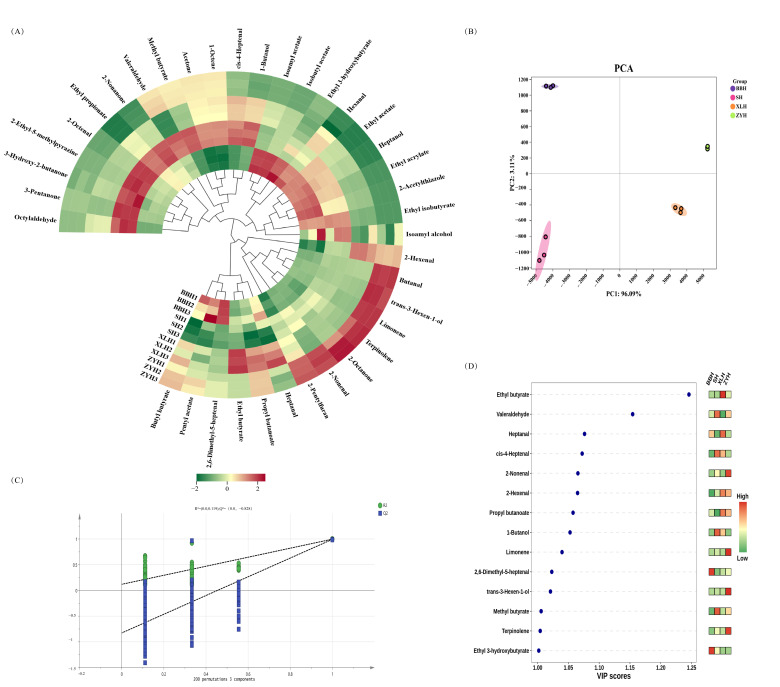
OAV analysis of VOCs with OAV ≥ 1 in four wine varieties. (**A**) Cluster heatmap of VOCs; (**B**) PCA of VOCs; (**C**) OPLS-DA permutation test (200 replacements); (**D**) VIP score plot (VIP > 1).

The four varieties differed in numbers of OAV ≥ 1 compounds: BBH (32), SH (35), XLH (34), and ZYH (36). ZYH had the lowest total volatile concentration but the highest number of OAV ≥ 1 compounds. This demonstrated that for these wines, aroma complexity depended on specific compounds rather than total concentration alone. PCA of the 37 OAV ≥ 1 compounds (Figure 6B) showed PC1 explaining 96.09% of variance and PC2 3.11%, confirming grape variety as the primary factor distinguishing aroma characteristics.

HCA was performed on 37 volatile compounds (OAV ≥ 1) from four wine varieties. Figure 6A reveals distinct OAV distribution patterns per variety, with specific compounds exhibiting significantly elevated OAVs. For instance, BBH showed higher OAVs for butyl butyrate, pentyl acetate, 2,6-dimethyl-5-heptenal, isoamyl acetate, and isobutyl acetate; SH for octanal, 3-pentanone, 3-hydroxy-2-butanone, and 2-ethyl-5-methylpyrazine; XLH for ethyl butyrate, heptanol, and propyl butanoate; and ZYH for 2-pentylfuran, 2-octanone, and 2-nonenal.

OPLS-DA of these compounds yielded a robust model (R^2^X = 0.963, R^2^Y = 0.994, Q^2^ = 0.987) with strong explanatory and predictive capacity. Permutation testing (n = 200, R^2^ = 0.105, Q^2^ = −0.852) confirmed no overfitting. VIP analysis identified 14 key compounds (VIP > 1): 1-butanol, 2,6-dimethyl-5-heptenal, 2-hexenal, 2-nonenal, cis-4-heptenal, ethyl 3-hydroxybutyrate, ethyl butyrate, heptanal, limonene, methyl butyrate, propyl butanoate, terpinolene, trans-3-hexen-1-ol, and valeraldehyde. Variations in their OAVs primarily explain aroma differences among varieties.

BBH’s key differential compounds 2,6-dimethyl-5-heptenal and ethyl 3-hydroxybutyrate showed significantly higher OAVs than other varieties, contributing green/fruity [37] and marshmallow-toasted nut aromas, likely due to specific fatty acid metabolism and yeast fermentation byproducts [38]. SH exhibited pronounced OAVs for valeraldehyde, 1-butanol, and cis-4-heptenal, imparting almond/bitter/malt/oil/pungent [39], fruity [40], and green/fruity [41] notes. Combined with higher phenolic content, these shaped its complex profile. XLH displayed elevated OAVs for ethyl butyrate, heptanal, 2-hexenal, and propyl butanoate, generating fruity [42], citrus/fat/green [43], and nut aromas. Enhanced ester synthesis may originate from grape juice precursors rich in acyl-CoA [44]. ZYH’s key compounds included 2-nonenal, terpinolene, trans-3-hexen-1-ol, and limonene. trans-3-Hexen-1-ol (green aroma) likely derives from the LOX pathway [45], while terpinolene and limonene contribute citrus/fresh/pine notes, potentially representing varietal-characteristic scents [46]. The inferred aroma characteristics are based on OAV calculations and literature-reported descriptors, lacking validation through consumer acceptance tests or professional sensory panels. Thus, direct correlations between these compounds and consumer preferences require future confirmation.

Analysis confirms varietal differences as the primary determinant of aroma profiles, consistent with prior research [47]. Xinjiang’s arid climate, high insolation, and large diurnal temperature variation promote accumulation of sugars, amino acids, and fatty acids in grapes [9]. This may influence metabolic pathways affecting aromatic compounds [48], mechanistically aligning with the Manas study on “high temperature/light regulating terpenoid and norisoprenoid accumulation” [14]. Collectively, these demonstrate Xinjiang’s terroir as a key environmental driver of wine aroma.

### 3.5. Comparative Analysis of Overall Aroma Profiles in Four Wine Varieties

E-Nose, a powerful tool for odor discrimination, enables objective assessment of the olfactory profiles of samples [49]. The response of its sensor array to the overall aroma profile shows high concordance with chemical analysis results. Radar chart analysis revealed that the BBH sample exhibited the highest response on sensor W5C (responsive to alkanes and aromatic components). Sensors W2S (responsive to alcohols, aldehydes, ketones), W1W (responsive to sulfides), W2W (responsive to aromatic compounds and sulfur-containing organics), and W1S (responsive to methane) showed their highest responses in the XLH sample. This aligns with HS-GC-IMS results detecting higher levels of aldehydes and ketones, such as heptanal and hexanal, in XLH. The SH sample displayed high responses on sensors W1C (responsive to methane and short-chain alkanes) and W3S (responsive to long-chain alkanes and aromatic compounds), potentially related to its unique pyrazines (e.g., 2-ethyl-5-methylpyrazine) and certain long-chain hydrocarbons. The prominent response of ZYH on sensor W5S (responsive to nitrogen oxides) may be associated with its aldehyde composition, warranting further investigation.

**Figure 7:**
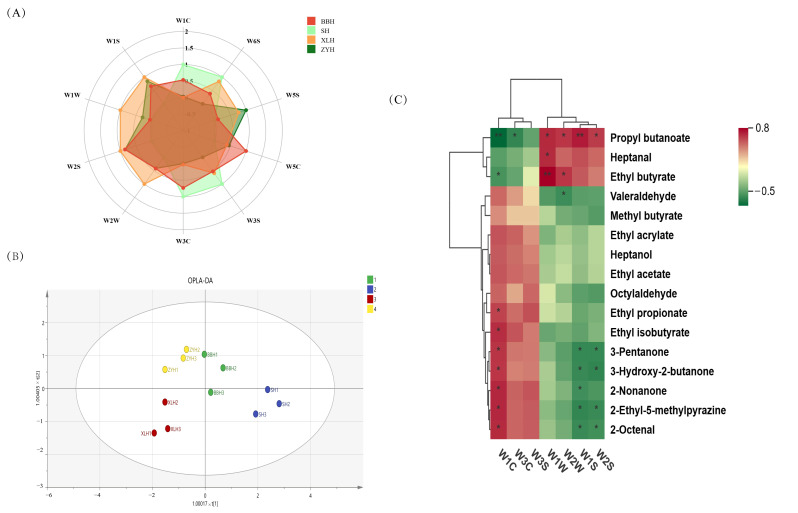
Electronic nose analysis of four wine varieties. (**A**) Radar chart of normalized sensor response scores; (**B**) OPLS-DA score plot of sensor-derived multivariate patterns; (**C**) Clustered heatmap of Pearson correlations between sensor responses and volatile compounds (OAV ≥ 1, * indicates *p* < 0.05, ** indicates *p* < 0.01).

OPLS-DA applied to the raw data effectively discriminated samples based on their properties (Figure 7B). The model demonstrated good fit and effectively distinguished the overall aroma profiles of the different Xinjiang *Vitis amurensis* wine varieties. Pearson correlation analysis (Figure 7C) statistically linked E-Nose sensor responses directly to volatile aroma compounds (OAV ≥ 1). For instance, sensors W2S, W2W, W1W, and W1S showed correlations with compounds such as propyl butanoate, ethyl butyrate, and heptanal, further explaining the strong response of XLH on these sensors. Responses from sensors W1C, W3C, and W3S exhibited significant positive correlations with compounds like 2-octenal, 2-ethyl-5-methylpyrazine, and 2-nonanone, confirming the basis for SH’s response pattern on these sensors. This correlation not only validates the reliability of the HS-GC-IMS data but also demonstrates that the E-Nose response patterns genuinely reflect true chemical compositional differences. This provides robust data support for the potential future use of E-Nose as a rapid detection tool for quality control of wines from this region.

## 4. Conclusions

This study employed a combined approach of HPLC, HS-GC-IMS, OAV calculation, electronic nose analysis, and multivariate statistical analysis to characterize, for the first time, the physicochemical properties and VOCs of wines produced from four *Vitis amurensis* varieties in Xinjiang, China.

Significant differences in physicochemical properties were observed among the varieties. The SH variety exhibited the highest levels of tannins (4.90 g/L), total phenols (3654.27 mg/L), and anthocyanins (545.40 mg/L), indicating superior antioxidant capacity and color intensity. Based on total sugar content, wines were classified as dry (BBH, XLH) or semi-dry (SH, ZYH). Total acidity also varied among varieties. Organic acid analysis identified tartaric acid, malic acid, and lactic acid as the predominant organic acids. Differences in the types and levels of organic acids influenced mouthfeel.

Regarding VOCs, total concentrations decreased in the order: BBH > SH > XLH > ZYH. Sixty-four volatile compounds were identified, with varying concentrations across the four varieties. Thirty-seven volatile aroma compounds had OAV ≥ 1. Ethyl isobutyrate, ethyl acetate, and cis-4-heptenal (OAV ≥ 1000) constitute core components contributing to fruitiness, sweetness, and dairy notes. VIP screening identified 14 key differential volatile aroma compounds (VIP > 1), whose OAV variations underlie inter-varietal flavor differences. Each variety exhibited distinct signature compounds: BBH featured 2,6-dimethyl-5-heptenal (green, fruity) and ethyl 3-hydroxybutyrate (marshmallow, toasted nuts); SH featured valeraldehyde (almond, bitter, malt, oil, pungent), 1-butanol (fruity), and cis-4-heptenal (green, fruity); XLH featured ethyl butyrate (fruity), heptanal (citrus, fat, green, nut), 2-hexenal (cheese), and propyl butanoate (apricot, fruit, pineapple, solvent); ZYH featured 2-nonenal (paper), terpinolene (pine), trans-3-hexen-1-ol (green), and limonene (citrus, fresh, pine). These compounds generate distinct odors, contributing to the unique flavor profile of each variety. Electronic nose sensor response patterns correlated highly with HS-GC-IMS results. For instance, the W2S sensor showed positive correlations with compounds such as propyl butanoate in XLH, supporting the combined future application of electronic nose and HS-GC-IMS technologies for exploring wine aroma characteristics.

In summary, this study constructed VOC fingerprint profiles for wines from four Xinjiang *Vitis amurensis* varieties, identifying key differentiating compounds. It provides theoretical support and data for enhancing regional wine quality from physicochemical and VOC perspectives, aiding in variety selection and development of distinctive regional wines. However, limitations include the lack of sensory evaluation to confirm the correlation between key compounds and consumer preference. Future studies should include a broader collection of wine samples, such as multiple vintages, and adopt more comprehensive analytical techniques, such as Gas Chromatography–Mass Spectrometry (GC-MS) and electronic tongue, to more fully reveal the flavor chemistry profile of Xinjiang *Vitis amurensis* wines.

## Figures and Tables

**Table 1:** Wine Sample Numbering Table.

Sample Number	Variety Name
BBH	Beibinghong
XLH	Xuelanhong
SH	Shuanghong
ZYH	Zuoyouhong

**Table 2:** E-Nose experimental conditions.

Parameter	Condition	Parameter	Condition
Vial	20 mL	Trap Initial Temp.	50 °C
Sample Amount	1 g/mL	Trap Split Flow	10 min
Incubation Temp.	50 °C	Trap Hold Time	34 s
Incubation Time	30 min	Trap Final Temp.	240 °C
Shaker Speed	500 r/min	Oven Initial Temp.	50 °C
Injection Volume	3000 μL	Temperature Program	2 °C/s to 250 °C
Injection Speed	125 μL/s	Acquisition Time	110 s
Inlet Temp.	200 °C	Detector Temp.	260 °C
Injection Duration	29 s	FID Gain	12

**Table 3:** Odor Activity Values (OAV) of Aroma Compounds in Four Wine Varieties.

**Compound**	Flavor Profile	Odor Threshold (µg/kg)	OAV > 1			
			BBH	SH	XLH	ZYH
Ethyl isobutyrate	Sweet, Fruit, Cheese	0.089	13,693.83 ± 209.89 b	14,060.92 ± 194.22 a	5985.70 ± 193.39 c	4393.84 ± 7.05 d
Ethyl acetate	Aromatic, Brandy, Grape	5	3325.01 ± 2.99 a	3063.88 ± 2.92 b	2568.70 ± 13.24 c	1870.44 ± 3.10 d
Ethyl butyrate	Apple, Butter, Cheese, Pineapple, Strawberry	0.9	1281.64 ± 91.78 b	1268.66 ± 73.52 b	1848.73 ± 14.15 a	1369.19 ± 1.45 b
Isoamyl acetate	Apple, Banana, Pear	19	193.68 ± 3.45 a	111.26 ± 2.15 c	128.72 ± 2.80 b	101.61 ± 0.36 d
Ethyl propionate	Apple, Pineapple, Rum, Strawberry	10	138.14 ± 0.98 b	195.89 ± 1.82 a	123.63 ± 2.14 c	27.18 ± 0.53 d
Ethyl acrylate	Pungent, Fragrant,	6.7	71.00 ± 2.18 a	52.74 ± 0.62 b	22.17 ± 0.67 c	7.96 ± 0.19 d
Isobutyl acetate	Apple, Banana, Floral, Herb	25	65.96 ± 1.16 a	21.63 ± 0.25 b	20.71 ± 0.48 b	7.36 ± 0.17 c
Propyl butanoate	Apricot, Fruit, Pineapple, Solvent	18	40.24 ± 0.53 c	38.43 ± 0.75 d	42.76 ± 0.38 a	41.84 ± 0.02 b
Pentyl acetate	Apple, Banana, Pear	43	35.73 ± 1.05 a	32.44 ± 0.65 c	33.43 ± 0.53 bc	34.28 ± 0.44 b
Butyl butyrate	Floral	400	1.21 ± 0.01 a	1.16 ± 0.00 b	1.21 ± 0.01 a	1.21 ± 0.00 a
Ethyl 3-hydroxybutyrate	Marshmallow, Roasted Nut	2500	1.89 ± 0.16 a	1.13 ± 0.20 b	0.81 ± 0.08 c	0.82 ± 0.04 c
Methyl butyrate	Apple, Banana, Cheese, Ester, Floral	59	0.88 ± 0.05 d	9.47 ± 0.12 a	3.01 ± 0.10 c	5.91 ± 0.10 b
Isoamyl alcohol	Burnt, Cocoa, Floral, Malt	4	108.92 ± 3.20 a	109.59 ± 1.85 a	106.15 ± 2.10 a	106.12 ± 1.86 a
Heptanol	Fat, Pungent	5.4	97.58 ± 3.14 a	81.00 ± 2.49 b	50.70 ± 1.35 c	19.06 ± 0.70 d
1-Butanol	Fruit	459.2	11.62 ± 0.03 c	13.34 ± 0.10 a	12.68 ± 0.03 b	11.59 ± 0.03 c
trans-3-Hexen-1-ol	Green	110	0.29 ± 0.04 c	0.45 ± 0.05 b	0.40 ± 0.04 bc	2.70 ± 0.14 a
cis-4-Heptenal	Green, Fruity	0.06	1015.82 ± 5.31 d	2611.15 ± 126.00 a	2218.63 ± 53.97 b	1386.48 ± 22.36 c
2-Nonenal	Paper	0.19	692.60 ± 11.73 c	963.96 ± 39.89 b	692.07 ± 14.07 c	1420.00 ± 20.49 a
Heptanal	Citrus, Fat, Green, Nut	2.8	466.62 ± 4.65 ab	447.31 ± 11.70 c	475.64 ± 5.25 a	454.02 ± 3.99 bc
2-Octenal	Dandelion, Fat, Fruit, Grass, Green, Spice	3	223.70 ± 6.62 b	451.24 ± 1.91 a	205.52 ± 1.86 c	137.64 ± 2.49 d
Octylaldehyde	Citrus, Fat, Green, Oil, Pungent	0.587	146.70 ± 7.71 d	1432.67 ± 114.32 a	565.29 ± 28.05 b	258.30 ± 3.95 c
2-Hexenal	Cheese	88.5	97.67 ± 1.59 d	104.57 ± 0.22 c	114.05 ± 0.90 a	111.18 ± 0.27 b
2,6-Dimethyl-5-heptenal	Fruit, Green, Melon	16	38.41 ± 0.46 a	9.62 ± 0.97 d	15.00 ± 0.52 c	19.01 ± 1.49 b
Hexanal	Apple, Fat, Fresh, Green, Oil	5	31.78 ± 1.46 a	24.29 ± 0.99 c	27.24 ± 0.93 b	16.38 ± 1.46 d
Valeraldehyde	Almond, Bitter, Malt, Oil, Pungent	12	6.65 ± 0.22 c	10.13 ± 0.32 a	4.64 ± 0.03 d	8.45 ± 0.19 b
Butanal	Banana, Green, Pungent	17	4.33 ± 0.52 c	7.27 ± 0.25 b	7.64 ± 0.11 b	25.80 ± 0.29 a
2-Acetylthiazole	Nut, Popcorn, Roast, Sulfur	3	234.79 ± 5.10 a	150.37 ± 1.70 b	87.57 ± 2.67 c	45.56 ± 0.95 d
2-Nonanone	Fragrant, Fruit, Green, Hot Milk	41	16.62 ± 0.72 b	25.58 ± 0.44 a	15.30 ± 0.08 c	9.45 ± 0.28 d
3-Pentanone	Green	40	10.02 ± 0.32 b	49.33 ± 0.35 a	7.96 ± 0.28 c	9.67 ± 0.23 b
3-Hydroxy-2-butanone	Butter, Creamy, Green Pepper	14	8.94 ± 0.49 bc	14.86 ± 0.69 a	9.51 ± 0.11 b	8.43 ± 0.34 c
2-Octanone	Fat, Fragrant, Mold	50.2	0.94 ± 0.04 c	1.33 ± 0.18 b	1.20 ± 0.01 bc	2.75 ± 0.40 a
Acetone	Pungent	832	0.72 ± 0.04 d	2.17 ± 0.03 a	1.72 ± 0.02 c	1.78 ± 0.01 b
Limonene	Citrus, Fresh, Pine	200	0.17 ± 0.02 c	0.30 ± 0.01 b	0.18 ± 0.01 c	1.25 ± 0.01 a
Terpinolene	Pine	200	0.53 ± 0.12 d	1.12 ± 0.10 b	0.83 ± 0.06 c	2.31 ± 0.20 a
1-Octene	Oil	0.5	449.79 ± 12.23 c	865.59 ± 6.27 a	741.50 ± 14.34 b	751.05 ± 4.98 b
2-Ethyl-5-methylpyrazine	Fruit, Green, Citrus,	100	2.63 ± 0.24 b	10.01 ± 0.44 a	2.17 ± 0.06 c	1.06 ± 0.04 d
2-Pentylfuran	Butter, Floral, Fruit, Green Bean	5.8	177.85 ± 4.61 d	203.56 ± 3.01 c	211.26 ± 4.44 b	238.86 ± 1.19 a

Notes: Means with different letters in the same column indicate significant differences (Duncan’s test, *p* < 0.05). Aroma descriptions from the website: https://www.femaflavor.org/; https://www.chemicalbook.com/ (accessed on 11 May 2025).

## Data Availability

The original contributions presented in the study are included in the article/Supplementary Materials. Further inquiries can be directed to the corresponding authors.

## Supplementary Materials

The following supporting information can be downloaded at https://www.mdpi.com/article/10.3390/foods14203521/s1, Table S1. Standard curves for six organic acids; Table S2. E-nose sensor information; Table S3. Composition of volatile compounds in four wine grape varieties.

## Abbreviations

The following abbreviations are used in this manuscript:

GC-MS	Gas Chromatography–Mass Spectrometry
HCA	Hierarchical cluster analysis
HPLC	High-Performance Liquid Chromatography
HS-GC-IMS	Headspace-Gas Chromatography–Ion Mobility Spectrometry
MLF	Malolactic fermentation
OAV	Odor Activity Value
OPLS-DA	Orthogonal Partial Least Squares-Discriminant Analysis
PCA	Principal component analysis
QDA	Quantitative descriptive analysis
VIP	Variable Importance in Projection
E-nose	Electronic nose
