# Supplementary material for: Analysis of Volatile Organic Compounds in Wines from Vitis amurensis Varieties in Xinjiang, China
Source: Foods. 2025 Oct 16;14(20):3521. doi: 10.3390/foods14203521 (PMC12562634; doi:10.3390/foods14203521)
Supplement: Supplementary file 1 [file foods-14-03521-s001.zip › foods-3861130-supplementary.pdf]

Supplementary Materials

Table S1. Standard curves for six organic acids.
Table S2. E-nose sensor information
Table S3. Composition of volatile compounds in four wine grape varieties

Table S1. Standard curves for six organic acids.

Compound	Linear range	Calibration curve	R^2
Tartaric acid	0.102-1.02 g/L	f(x) = 2366.4x + 0.9989	1
Malic acid	0.1095-1.095 g/L	f(x) = 1239.1x - 4.3401	0.9999
Lactic acid	0.092-0.92 g/L	f(x) = 1123.8x - 7.0687	0.9999
Glacial acetic acid	1-10g/L	f(x) = 832.47x - 6.3193	1
Citric acid	0.102-1.02 g/L	f(x) = 1548.9x - 18.395	0.9998
Succinic acid	0.109-1.09 g/L	f(x) = 729.12x - 14.285	0.9999

Table S2. E-nose sensor information.

Sensor	Performance description
W1C	Aromatic compounds
W5S	Broad-range compounds, polar compounds, nitrogen oxides and ozone
W3C	Ammonia, aromatic compounds, aldehydes and ketones
W6S	Hydrogen and broad-range compounds
W5C	Arom-aliph, alkanes, aromatic compounds and less polar compounds
W1S	Methane, broadmethane and broad-range compounds
W1W	Sulfur compounds, terpenes and sulfur organic compounds
W2S	Alcohols, partially aromatic compounds and ketones
W2W	Aromatic compounds and sulfur organic compounds
W3S	Methane-aliph

Table S3. Composition of volatile compounds in four wine grape varieties.

CAS#	Substance Name	Volatile Compound Content（μg·L^{-1}）			
		**BBH**	**SH**	**XLH**	**ZYH**
71-36-3	1-Butanol	5335.62±12.2c	6126.41±46.64a	5820.48±13.04b	5321.03±13.75c
71-41-0	1-Pentanol	56.17±2.17c	110.57±3.90a	61.88±1.59b	55.23±3.27c
98-85-1	1-Phenylethanol	117.02±4.84b	200.50±4.99a	90.98±1.67c	89.62±3.11c
111-76-2	2-Butoxyethanol	158.58±4.35d	173.43±3.21c	192.66±2.14b	214.39±2.16a
626-93-7	2-Hexanol	319.88±9.99a	246.65±11.85c	266.67±0.58b	96.87±2.32d
763-32-6	3-Methyl-3-buten-1-ol	99.85±6.75c	309.36±17.36a	200.77±7.04b	106.85±4.52c
584-02-1	3-Pentanol	957.91±16.87a	851.62±8.70c	936.78±1.57b	688.01±2.93d
626-89-1	4-Methyl-1-pentanol	59.02±3.95a	10.68±0.29b	4.41±0.13c	4.06±0.17c
3857-25-8	5-Methylfurfuryl alcohol	394.82±1.64b	420.98±6.16a	340.1±1.36d	368.63±1.91c
111-70-6	Heptanol	526.91±16.93a	437.38±13.44b	273.76±7.31c	102.95±3.78d
123-51-3	Isoamyl alcohol	435.67±12.82a	438.36±7.39a	424.6±8.40a	424.50±7.44a
928-97-2	trans-3-Hexen-1-ol	31.82±4.50c	49.65±4.95b	43.66±4.07bc	297.41±14.94a
**Alcohols**	**Subtotal**	**8493.26**	**9375.60**	**8656.76**	**7769.54**
**12**	**Percentage**	**11.78%**	**13.29%**	**14.23%**	**14.34%**
6728-26-3	2-Hexenal	8643.95±140.50d	9254.58±19.70c	10093.24±79.74a	9839.14±23.48b
18829-56-6	2-Nonenal	131.59±2.23c	183.15±7.58b	131.49±2.67c	269.80±3.89a
111-71-7	Heptanal	1306.53±13.02ab	1252.46±32.77c	1331.78±14.70a	1271.26±11.18bc
66-25-1	Hexanal	158.92±7.32a	121.43±4.93c	136.20±4.67b	81.90±7.28d
124-13-0	Octylaldehyde	86.11±4.53d	840.98±67.11a	331.83±16.47b	151.62±2.32c
110-62-3	Valeraldehyde	79.78±2.58c	121.59±3.82a	55.63±0.40d	101.39±2.27b
623-36-9	2-Methyl-2-pentenal	8.01±1.26b	7.56±0.35b	7.79±0.66b	54.87±2.68a
123-72-8	Butanal	73.69±8.91c	123.64±4.17b	129.92±1.85b	438.63±4.97a
106-72-9	2,6-Dimethyl-5-heptenal	614.50±7.35a	153.87±15.54d	239.95±8.36c	304.09±23.83b

Table S3.Cont

CAS#	Substance Name	Volatile Compound Content（$\mu g \cdot L^{-1}$）			
		BBH	SH	XLH	ZYH
2548-87-0	2-Octenal	671.10±19.85b	1353.71±5.74a	616.55±5.59c	412.92±7.46d
107-86-8	3-Methyl-2-butenal	93.29±7.13c	112.86±5.48b	35.33±1.53d	446.48±7.94a
6728-31-0	cis-4-Heptenal	60.95±0.32d	156.67±7.56a	133.12±3.24b	83.19±1.34c
**Aldehydes**	**Subtotal**	**11928.42**	**13682.50**	**13242.83**	**13455.28**
**12**	**Percentage**	**16.54%**	**19.39%**	**21.77%**	**24.83%**
108-83-8	2,6-Dimethyl-4-heptanone	285.72±2.74c	311.81±4.48b	353.92±5.91a	199.09±6.99d
821-55-6	2-Nonanone	681.34±29.63b	1048.74±18.10a	627.39±3.10c	387.39±11.29d
111-13-7	2-Octanone	47.15±2.17b	66.89±8.79b	60.22±0.58b	137.86±20.11a
96-22-0	3-Pentanone	400.87±12.72b	1973.04±14.02a	318.26±11.06c	386.87±9.16b
67-64-1	Acetone	599.65±33.56d	1808.74±27.61a	1432.26±19.38b	1482.61±11.07c
78-59-1	Isophorone	1236.24±22.42c	1299.50±17.67b	1468.18±4.69a	1271.69±7.70b
24295-03-2	2-Acetylthiazole	704.36±15.31a	451.11±5.10b	262.70±8.01c	136.69±2.85d
513-86-0	3-Hydroxy-2-butanone	125.10±6.85bc	208.04±9.67a	133.12±1.60b	118.05±4.75d
120-92-3	Cyclopentanone	227.94±10.33a	185.66±2.78b	103.57±3.70d	140.40±3.30c
**Ketones**	**Subtotal**	**4308.37**	**7353.52**	**4759.61**	**4260.65**
**9**	**Percentage**	**5.97%**	**10.42%**	**7.82%**	**7.86%**
109-21-7	Butyl butyrate	484.67±5.70a	465.34±1.87b	482.24±3.60a	483.43±1.99a
109-19-3	Butyl isovalerate	259.51±2.50c	325.74±4.80b	443.35±2.11a	243.89±3.77d
27829-72-7	Ethyl 2-hexenoate	270.18±89.53a	344.56±222.06a	239.72±51.07a	159.55±3.09a
5405-41-4	Ethyl 3-hydroxybutyrate	4724.84±408.55a	2825.36±509.81b	2025.31±201.59c	2051.77±89.77c
141-78-6	Ethyl acetate	16625.03±14.94a	15319.41±14.58b	12843.5±66.22c	9352.22±15.49d
140-88-5	Ethyl acrylate	475.70±14.60a	353.33±4.17b	148.52±4.51c	53.30±1.26d
105-54-4	Ethyl butyrate-M	1003.82±81.85a	901.03±62.89a	776.66±25.87b	989.01±3.18a

Table S3.Cont

CAS#	Substance Name	Volatile Compound Content（μg·L^{-1}）			
		**BBH**	**SH**	**XLH**	**ZYH**
105-54-4	Ethyl butyrate-D	149.66±5.74c	240.76±6.59b	887.20±20.59a	243.26±1.91b
623-70-1	Ethyl crotonate	11.24±1.44c	11.55±0.63c	13.95±0.63b	84.87±1.46a
109-94-4	Ethyl formate	82.64±4.4c	100.31±4.46b	160.17±3.65a	97.64±1.86b
97-62-1	Ethyl isobutyrate	1218.75±18.68b	1251.42±17.29a	532.73±17.21c	391.05±0.63d
97-64-3	Ethyl lactate-M	1563.95±6.00a	779.77±28.05c	994.32±26.53b	1012.94±26.9b
97-64-3	Ethyl lactate-D	1846.99±157.83a	102.77±13.41b	154.57±8.07b	247.78±34.39b
105-37-3	Ethyl propionate	1381.41±9.83b	1958.94±18.24a	1236.28±21.40c	271.82±5.34d
123-92-2	Isoamyl acetate	3679.89±65.54a	2113.93±40.79c	2445.69±53.23b	1930.67±6.88d
110-45-2	Isoamyl formate	1931.02±11.34a	1767.59±10.41b	1435.00±6.92d	1609.62±0.85c
110-19-0	Isobutyl acetate	1649.00±28.99a	540.64±6.20b	517.68±12.05b	183.96±4.14c
540-42-1	Isobutyl propanoate	18.74±1.24d	50.55±1.57c	144.11±3.70a	77.41±0.92b
540-42-1	Isobutyl propionate	103.04±2.45a	94.74±5.03b	92.22±2.07b	74.65±0.96c
79-20-9	Methyl acetate	4100.79±43.66a	3751.79±15.40b	2981.43±11.80c	2982.61±16.69c
623-42-7	Methyl butyrate	52.10±2.99d	558.49±7.07a	177.58±5.94c	348.84±5.72b
628-63-7	Pentyl acetate	1536.31±44.95a	1394.79±27.86c	1437.61±22.84bc	1474.25±19b
109-60-4	Propyl acetate	752.30±6.48a	322.69±6.77b	183.83±7.36c	51.57±1.11d
105-66-8	Propyl butanoate	724.23±9.51b	691.69±13.55c	769.67±6.84a	753.15±0.32a
106-36-5	Propyl propionate	8.25±1.02d	49.24±1.68b	17.30±0.46c	67.45±1.14a
**Esters**	**Subtotal**	**44654.05**	**36316.43**	**31140.64**	**25236.70**
**23**	**Percentage**	**61.92%**	**51.47%**	**51.19%**	**46.57%**
111-66-0	1-Octene	224.90±6.12c	432.79±3.13a	370.75±7.17b	375.53±2.49b
138-86-3	Limonene	33.65±3.11c	60.43±1.61b	35.35±1.22c	249.03±1.42a
586-62-9	Terpinolene	106.52±23.68d	224.62±19.15b	166.85±12.47c	461.02±39.15a

Table S3.Cont

CAS#	Substance Name	Volatile Compound Content（μg·L^{-1}）			
		BBH	SH	XLH	ZYH
**Alkenes**	**Subtotal**	**365.07**	**717.84**	**572.95**	**1085.58**
**3**	**Percentage**	**0.51%**	**1.02%**	**0.94%**	**2.00%**
97-61-0	2-Methylvaleric acid	538.48±9.10b	487.51±26.02c	630.22±7.67a	413.42±1.65d
107-92-6	Butyric acid	451.01±14.63a	400.12±2.82b	363.09±8.56c	455.39±1.62a
**Acids**	**Subtotal**	**989.49**	**887.63**	**993.31**	**868.81**
**2**	**Percentage**	**1.37%**	**1.26%**	**1.63%**	**1.60%**
13360-64-0	2-Ethyl-5-methylpyrazine	263.04±23.61b	1001.32±43.68a	216.95±5.88b	105.87±4.24c
**Pyrazines**	**Subtotal**	**263.04**	**1001.32**	**216.95**	**105.87**
**1**	**Percentage**	**0.36%**	**1.42%**	**0.36%**	**0.20%**
1072-83-9	2-Acetylpyrrole	78.20±3.81a	47.02±3.32b	27.89±2.00c	19.56±3.22d
3777-69-3	2-Pentylfuran	1031.51±26.74d	1180.64±17.46c	1225.30±25.77b	1385.38±6.89a
**Others**	**Subtotal**	**1109.71**	**1227.66**	**1253.18**	**1404.95**
**2**	**Percentage**	**1.54%**	**1.74%**	**2.06%**	**2.59%**
	**Total**	**72111.42**	**70562.50**	**60836.22**	**54187.37**

Different lowercase letters indicate significant differences between treatments. (Duncan's test $p < 0.05$).

-M and -D, which are the Monomer and Dimer of the same substance.
